# Bioactive dietary polyphenols modulate hippocampal inflammasome activation and sex-specific affective behavioral deficits following peripheral Foxp3^+^ regulatory T cell depletion

**DOI:** 10.21203/rs.3.rs-10425197/v1

**Published:** 2026-08-24

**Authors:** Eun-Jeong Yang, Hsiao-Yun Lin, Johana Alvarez, Scott J Russo, Carlos Toro-Chacon, Giulio Maria Pasinetti

**Affiliations:** 1 Department of Neurology, Icahn School of Medicine at Mount Sinai, New York, NY, United States; 2 Nash Family Department of Neuroscience, Icahn School of Medicine at Mount Sinai, New York, NY, USA.; 3 Brain-Body Research Institute, Icahn School of Medicine at Mount Sinai, New York, NY, USA.; 4 Friedman Brain Institute, Icahn School of Medicine at Mount Sinai, New York, NY, USA.; 5 Spinal Cord Damage Research Center, James J. Peters VA Medical Center, Bronx, NY, USA.; 6 VA Sequencing Collaborations United for Research and Epidemiology (seqCURE), James J. Peters VA Medical Center, Bronx, NY, USA.; 7 The Bronx Veterans Medical Research Foundation, Bronx, NY, USA.; 8 James J. Peters VA Medical Center, Geriatrics Research, Education and Clinical Center (GRECC), Bronx, NY, USA.

**Keywords:** Foxp3, regulatory T cells, BDPP, inflammasome, Caspase-1, microglia, neuroimmune axis, sex differences

## Abstract

The transcription factor Foxp3 is the lineage-defining marker essential for the development and suppressive function of regulatory T cells (Tregs), which maintain peripheral immune homeostasis. Emerging evidence suggests that this Foxp3+ Treg axis plays a critical role in modulating central neuroimmune signaling and affective behaviors; however, whether a Bioactive Dietary Polyphenol Preparation (BDPP) can effectively modulate or attenuate these neuroinflammatory responses remains unclear. In this study, we assessed how transient Foxp3 depletion influences affective behaviors, microglial responses, and blood–brain barrier integrity within the hippocampus, and whether BDPP treatment could modulate these outcomes. Foxp3 depletion induced sex-dependent behavioral deficits, characterized by depressive-like behavior, impaired motivational drive, and anxiety-related behavior. These behavioral changes coincided with robust hippocampal Caspase-1 activation and microglial remodeling, as quantified by Sholl analysis, indicating an enhanced inflammasome-associated neuroimmune response following peripheral Treg dysfunction. Notably, BBB integrity was preserved across all groups, demonstrating that peripheral immune dysregulation is sufficient to drive central neuroinflammatory and behavioral abnormalities independent of vascular damage. Furthermore, BDPP administration selectively attenuated these behavioral deficits, caspase-1 activation, and the associated microglial changes predominantly in male subjects, whereas females exhibited limited responsiveness. Collectively, these data establish a functional link between peripheral Treg homeostasis and hippocampal immunity, highlighting BDPP as a sex-specific modulator of immune-driven behavioral dysfunction.

## Introduction

Regulatory T cells (Tregs) are a specialized subset of CD4^+^ T cells required for the maintenance of immune homeostasis and self-tolerance (1). Their suppressive function is critically dependent on the transcription factor Foxp3 (forkhead box protein 3), which governs Treg lineage stability and immunoregulatory capacity (1). Genetic or functional disruption of Foxp3 results in uncontrolled immune activation and severe systemic autoimmunity, underscoring the indispensable role of Tregs in immune regulation (2). These findings establish Tregs as central modulators of immune balance under both physiological and pathological conditions (1, 2). Beyond their peripheral immune function, accumulating evidence indicates that Tregs also participate in regulating neuroimmune interactions (3). Peripheral immune signals are increasingly recognized as key modulators of central nervous system (CNS) function, influencing synaptic plasticity, neuroinflammation, and behavioral outputs (4, 5). However, it remains unclear whether Treg dysfunction affects distinct neurobehavioral domains, such as affective, motivational, and anxiety-related behaviors. In addition, the molecular pathways linking peripheral Treg dysfunction to central brain responses remain poorly defined, particularly in the hippocampus, which is highly susceptible to systemic immune perturbation and critical in regulating mood-related behaviors (6–8).

The hippocampus is particularly vulnerable to immune-mediated perturbation due to its high density of microglia and distinct sensitivity to systemic inflammatory signals (8–10). Stress-associated immune activation preferentially affects hippocampal and prefrontal circuits, suggesting circuit-specific vulnerability to immune perturbation (11–13). Microglia are recognized as key mediators of immune-to-brain communication and are dynamically regulated by peripheral immune status, including T cell–derived signals (14). Inflammasome signaling has emerged as a critical mechanism linking innate immune activation to synaptic and behavioral dysfunction in microglia (15). In particular, activation of the NLRP3 inflammasome is implicated in stress-related behavioral phenotypes and synaptic dysfunction in limbic circuits (16). As the brain’s principal innate immune cells, microglia mediate inflammasome activity and serve as functional interfaces between peripheral immune signals and neuronal circuits (17). These findings suggest a functional coupling between Treg activity, microglial state, and inflammasome signaling in regulating brain immune tone.

Dietary polyphenols have emerged as promising modulators of immune and neuroinflammatory processes (18). Polyphenol-rich preparations influence inflammatory signaling, microglial activation, and behavioral outcomes in models of neuropsychiatric and neurodegenerative disorders (19). However, despite the therapeutic potential of isolated polyphenols, their clinical translation is hindered by poor bioavailability and rapid metabolic clearance (20). Most dietary polyphenols undergo extensive degradation in the gastrointestinal tract, resulting in insufficient systemic exposure and minimal penetration across the blood-brain barrier (BBB) (21). To address these pharmacokinetic limitations, we have previously characterized a Bioactive Dietary Polyphenol Preparation (BDPP)—a synergistic formulation composed of grape seed polyphenol preparation (GSPE), Concord grape juice (CGJ), and resveratrol (RSV) (22). The BDPP formulation serve as a superior alternative, employing a synergistic multi-component strategy that optimizes the generation of brain-permeable metabolites (22, 23). Specifically, BDPP has been shown to significantly increase the systemic concentration of bioactive compounds, such as 3-hydroxyphenylpropionic acid and malvidin-glucoside, which effectively cross the BBB to modulate neuroimmune responses (24, 25). Accordingly, BDPP provides a strategic pharmacological platform to examine whether the central neuroimmune consequences of Treg dysfunction can be modulated through specific inflammatory signaling pathways.

Integrating sex as a biological variable is imperative for a nuanced understanding of mood-related disorders, given the well-established sexual dimorphism in immune responses and neuroinflammatory signaling (26). Despite these well-documented sex differences, many neuroimmune studies remain sex-biased, thereby overlooking sex-dependent regulatory mechanisms of immune–brain interactions, including microglial function (27–29). Notably, sex-specific sensitivities particularly within microglial signaling pathways, suggest that behavioral vulnerability to Treg depletion, and subsequent responsiveness to BDPP, may arise from distinct underlying biological mechanisms in males and females. Accordingly, incorporating sex as a biological variable is essential for accurately defining how BDPP restores immune and neural homeostasis across divergent physiological contexts.

In this study, we utilize a transient Foxp3-depletion model to characterize the behavioral consequences of peripheral Treg dysfunction across affective, motivational, and anxiety-related domains in both male and female mice. We specifically investigate whether hippocampal inflammasome activation and microglial remodeling underlie these behavioral alterations as key neuroimmune mechanisms. In parallel, we assess the therapeutic potential of BDPP to attenuate these neuroimmune and behavioral phenotypes, and determine its efficacy in a sex-dependent manner.

## Materials and methods

### Animals

All experimental procedures were conducted in accordance with protocols approved by the Mount Sinai Institutional Animal Care and Use Committee (IACUC; protocol number: PROTO202000019). Male or female Foxp3 conditional knockout (cKO) mice expressing a diphtheria toxin receptor (DTR)-enhanced GFP transgene under the control of the Foxp3 promoter (C57BL/6-Tg(Foxp3-DTR/EGFP)23.2Spar/Mmjax) were obtained from The Jackson Laboratory. These mice were maintained by breeding with C57BL/6J mice, and wild-type (WT) controls were derived as littermates from the same breeding pairs. Genotyping of transgenic offspring was performed by PCR using the following primers: forward, 5′-CCCAGGTTACCATGGAGAGA-3′; reverse, 5′-GAACTTCAGGGTCAGCTTGC-3′. Non-transgenic littermates were used as age-matched controls. Both heterozygous Foxp3 cKO mice and WT littermates were included in all experiments. All animals were housed under a 12 h light/dark cycle in a temperature-controlled environment.

### Administration of diphtheria toxin (DT)

To establish an optimal dosing regimen for diphtheria toxin (DT; D0564, Sigma-Aldrich), Foxp3 conditional knockout (cKO) mice received intraperitoneal (i.p.) injections of 300 ng DT or phosphate-buffered saline (PBS) as a control every 4 days for 6 weeks. This regimen was selected to deplete Foxp3^+^ cells while minimizing potential nonspecific toxic effects efficiently (30).

### Administration of BDPP

BDPP was administered via drinking water for a total of 7 weeks, beginning two weeks prior to DT injection and continuing throughout the entire DT administration and behavioral testing period. The preparation consisted of three components delivered simultaneously: grape seed polyphenol extract (GSPE) at 200 mg/kg/day, resveratrol (RSV) at 300 mg/kg/day, and Concord grape juice (CGJ) providing a total polyphenol content of 183 mg/kg/day (31). Dosing was established by scaling from estimated human dietary polyphenol consumption of approximately 1g/day, of which phenolic acids constitute roughly 30%, yielding an effective intake of 330mg/day (32). Interspecies dose conversion was performed using the body surface area normalization formula recommended by the USDA, which yields a mouse-equivalent dose of approximately 62 mg/kg/day of total polyphenols. Individual component doses were further informed by prior studies demonstrating behavioral and neuroimmune efficacy of each constituent in rodent models. Drinking solutions were prepared fresh weekly, and fluid intake was monitored throughout to ensure consistent delivery. Body weight was recorded weekly across all experimental groups to verify that BDPP administration did not induce metabolic alterations.

### Behavioral tests

Behavioral assessments were conducted using a near-infrared camera system and analyzed with ANY-maze^™^ tracking software (Stoelting, IL, USA). Before testing, all mice were habituated to daily handling for 10 min over 5 consecutive days and acclimated to the testing room for 1 h before the start of experiments. All behavioral paradigms were performed according to standard procedures, as summarized below.

Open field test (OF): Locomotor activity was evaluated by placing each mouse in a white open-field arena (42.5 cm × 42.5 cm × 42.5 cm) for 10 min. Total distance traveled was automatically quantified using ANY-maze software.

Light and dark box test (LD): Anxiety-like behavior was assessed using a two-compartment light–dark apparatus (each compartment: 20 cm × 40 cm × 40 cm). Mice were allowed to explore the apparatus freely for 10 min, and behavior was recorded with an overhead camera. Time spent in the dark compartment was analyzed using ANY-maze automated tracking.

Forced swimming test (FST): Depressive-like behavior was assessed by placing mice in a transparent cylindrical container (45 cm height, 20 cm diameter) filled with water. Each session was recorded for 6 min, and immobility time—defined as the absence of active limb movements—was quantified using ANY-maze software.

Nest-building test: With reference to previous reports (33), nest building was assessed 24 hours after the introduction of nesting materials. Scoring was performed by quantifying the area of the nest formed within the cage. The nest score was calculated based on the proportion of the cage area occupied by the nest (nest area/total cage area).

### Tissue preparation

For brain tissue collection, mice from all experimental groups were sacrificed following the completion of behavioral testing. Briefly, mice were deeply anesthetized with ketamine/xylazine (100 mg/kg and 5 mg/kg, respectively, via intraperitoneal injection). Following confirmation of surgical anesthesia by the absence of a toe-pinch reflex, animals were euthanized by exsanguination during transcardial perfusion with PBS. Brains were then harvested for downstream analyses. Brains were hemisected, and each hemisphere was processed separately. One hemisphere was dissected to isolate the hippocampal formation, while the contralateral hemisphere was fixed in 4% paraformaldehyde (PFA) for 24 hours. All samples were subsequently stored at −80 °C or 4 °C until further analysis.

### Western blotting

Protein lysates extracted from the hippocampus (45 μg per sample) were resolved on 4–15% sodium dodecyl sulfate–polyacrylamide gels and transferred onto nitrocellulose membranes. Membranes were blocked for 1 h at room temperature (RT) and then incubated overnight at 4 °C with primary antibodies against Caspase-1 (20B-0042). The following day, membranes were incubated for 1 h at RT with horseradish peroxidase (HRP)-conjugated secondary antibodies (anti-rabbit or anti-mouse IgG). Immunoreactive bands were visualized using a chemiluminescence detection kit (1705061; Bio-Rad). To re-probe for the loading control, bound antibodies were removed by incubating the membranes in Restore^™^ Western Blot Stripping Buffer (46430; Thermo Fisher Scientific) for 15 min at RT. Membranes were then washed thoroughly with TBST, blocked for 1 h at RT, and re-probed overnight at 4°C with primary antibodies against beta-actin (GTX109639). Following incubation with HRP-conjugated secondary antibodies and chemiluminescent detection, band intensities were quantified using ImageJ software, and relative protein expression levels were normalized to beta-actin.

### Immunohistochemistry

Brains (N = 3–5 per group) were post-fixed in 4% paraformaldehyde (PFA) and subsequently equilibrated in PBS. Coronal brain sections (50 μm) were washed with PBS, permeabilized in PBS containing 0.2% Triton X-100 (PBST), and blocked with 2% normal goat serum in PBST. Sections were incubated overnight at 4 °C with anti-ionized calcium-binding adaptor molecule 1 (Iba1; 1:1000, ab178846, Abcam, MA, USA) diluted in PBST containing 2% normal goat serum. After washing, sections were incubated for 1 h at room temperature with Alexa Fluor 568-conjugated secondary antibody (1:500 in PBST containing 2% normal goat serum). Following additional washes, sections were mounted onto microscope slides and coverslipped. Images were acquired using a Zeiss LSM 880 confocal microscope (Zeiss, Germany). Microscopy and image analyses were performed at the Microscopy CoRE at the Icahn School of Medicine at Mount Sinai.

### Sholl analysis

To assess microglial morphological complexity, a Sholl analysis was conducted (34). Quantitative analysis of microglial morphology in the hippocampus was performed using IMARIS version 9.1.2 (Bitplane Inc., MA, USA). Parameters analyzed included soma volume, total branch length, number of terminal points, and number of intersection segments. For three-dimensional reconstruction, z-stack confocal images were first deconvolved using AutoQuant X3.1 (Media Cybernetics, MD, USA) to reduce optical blur and then imported into IMARIS for analysis. The Filament module was used to reconstruct microglial cell bodies and processes. One representative microglial cell was selected from each imaging field, and the soma and processes were manually traced to generate a three-dimensional model. Quantification included the number of primary processes and branch termini. Sholl analysis was performed by placing concentric circles centered on the soma at 5 μm intervals and counting the number of process intersections at each radius. Soma volume, branch length, terminal points, and intersection counts were measured for each cell, and mean values were calculated for each experimental group. A total of 3–5 mice per group were analyzed, with 3–4 tissue sections per mouse and 4–15 microglia examined per section.

### BBB permeability assay

Following completion of all behavioral experiments, BBB permeability was assessed using Evans Blue dye extravasation as previously validated (35, 36). Mice were intravenously injected via the retro-orbital sinus with 2% Evans Blue solution (Sigma-Aldrich, 314136) diluted in sterile PBS(200 μL per mouse). After 16 hours of circulation, animals were deeply anesthetized and transcardially perfused with ice-cold normal saline to remove intravascular dye. Brains were then rapidly extracted and processed to quantify Evans Blue extravasation. Briefly, brain tissues were homogenized and incubated in dimethylformamide at 55 °C for 48 hours to extract the dye. Following incubation, samples were centrifuged at 21,000 × g for 30 minutes at room temperature. The supernatant was collected, and Evans Blue concentration was determined by measuring absorbance at 620nm with a plate reader. Dye content was calculated against a standard curve and normalized to tissue weight.

### Statistical analysis

All data are expressed as the mean ± standard error of the mean. Data were analyzed using two-way analysis of variance (ANOVA) followed by Tukey’s post hoc analysis (*p < 0.05, **p < 0.01, ***p<0.001). All statistical analyses were performed using GraphPad Prism 10 software (GraphPad Software Inc., San Diego, CA, USA).

## Results

### Peripheral Foxp3+ Tregs depletion induces affective and motivational deficits that are effectively rescued by BDPP

**Figure 1.**
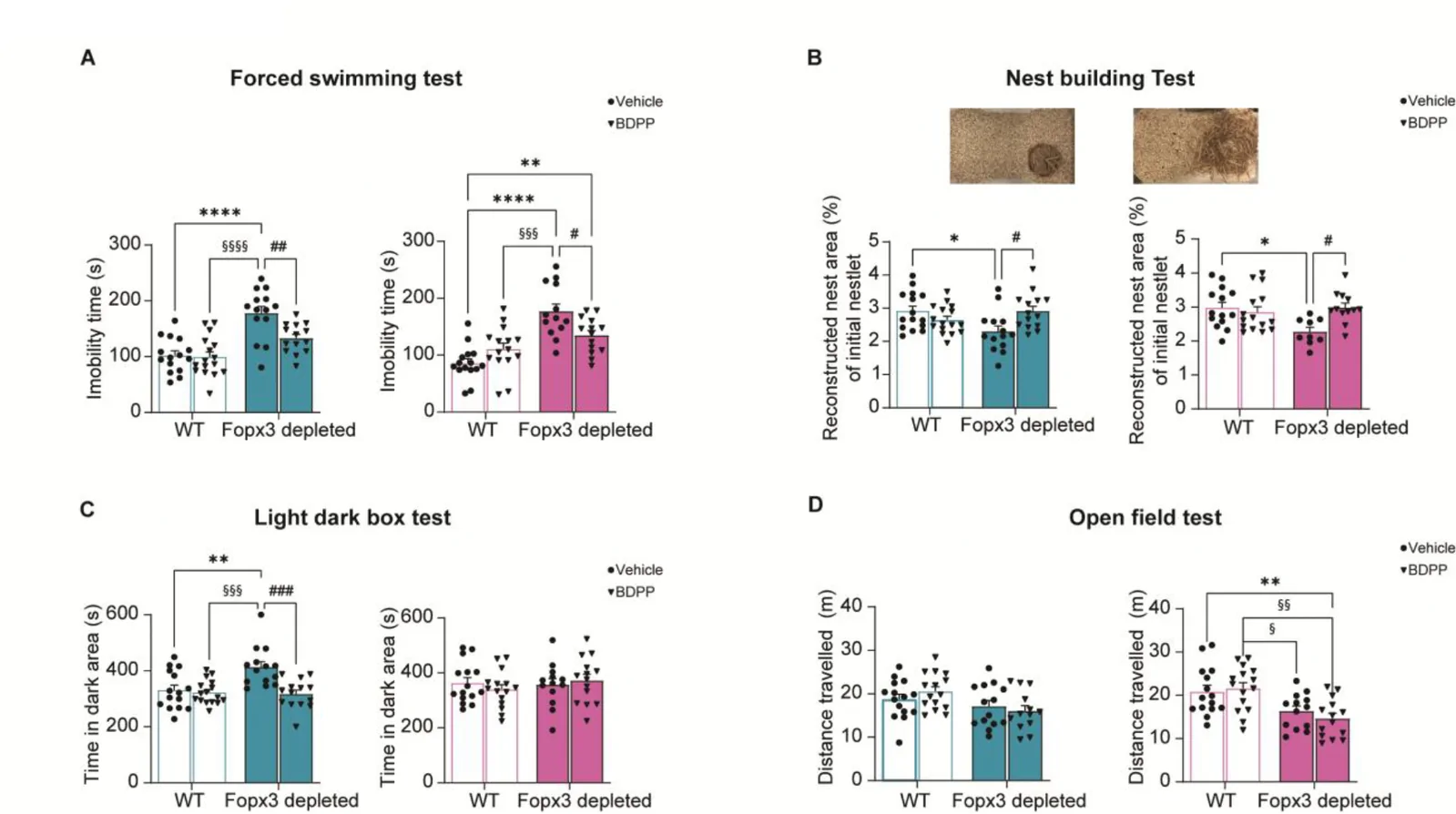
Behavioral effects of Foxp3+ Tregs depletion and BDPP treatment across affective and motivational assays. (A) Bar graphs showing total immobility time as a measure of depression-like behavior in forced swimming test (FST). (B) Bar graphs showing reconstructed nest area as a measure of motivational drive and self-care behavior in the nest-building test. (C) Bar graphs showing time spent in the dark compartment as a measure of anxiety-like phenotypes in the light–dark (LD) box. (D) Bar graphs showing total distance traveled as a measure of locomotor vigor in the open field test (OF). Data are presented as mean ± SEM. WT groups are indicated by white bars with blue (males) or pink (females) borders, while Foxp3-depleted males and females are shown as solid blue and pink bars, respectively. Individual subjects are represented as circles (vehicle-treated) or inverted triangles (BDPP-treated). Statistical analyses were performed using two-way ANOVA followed by Tukey’s post hoc test (*p<0.05; **p<0.01; ****p<0.0001; compared with age-matched vehicle-treated WT mice, ^§^p<0.05; ^§§^p<0.01 ^§§§^p<0.001; ^§§§§^p<0.0001; compared with age-matched BDPP-treated WT mice, ^#^p<0.05; ^##^p<0.01; ^###^p<0.001; compared with age-matched vehicle-treated Foxp3-depleted mice, n = 13–22 mice for each group).

To elucidate the role of Foxp3+ Tregs in maintaining affective and motivational homeostasis, we utilized a well-established transient depletion model in Foxp3-cKO mice. We have previously characterized and validated the efficacy of this specific dosing regimen (300 ng of DT every 4 days for 6 weeks), demonstrating a significant and selective reduction of Foxp3-expressing cells by approximately 60% in peripheral blood mononuclear cells and 46% in the spleen, without altering baseline Foxp3 expression within the brain parenchyma (30). Based on this robust and peripheral-selective depletion profile, we performed comprehensive multidimensional behavioral phenotyping in Foxp3-depleted mice encompassing a broad spectrum of distinct neuropsychiatric domains (Figure 1 and Supplementary Figure 1A). Foxp3 depletion and BDPP treatment did not significantly affect body weight, food intake, or water consumption throughout the experimental period (Supplementary Figure 1B), demonstrating that the observed behavioral phenotypes were independent of generalized metabolic confounding factors. In the FST, vehicle-treated Foxp3-depleted mice of both sexes exhibited significantly increased immobility time compared to vehicle-treated WT mice (Figure 1A; ****p < 0.0001), indicative of depressive-like behavior. Notably, BDPP treatment significantly attenuated this deficit, reducing immobility in both male and female groups (Figure 1A; ^##^p < 0.01, ^#^p < 0.05). This neurobehavioral decline extended to motivational deficits, as assessed by the nest-building test. Vehicle-treated Foxp3-depleted mice displayed a substantial reduction in reconstructed nest area (Figure 1B; *p < 0.05), indicating impaired goal-directed behavior. Consistent with its behavioral efficacy, BDPP administration effectively restored nesting performance to WT baseline levels (Figure 1B; ^#^p < 0.05). Interestingly, the neurobehavioral impacts of Foxp3 depletion exhibited distinct sexual dimorphism regarding anxiety-like behavior and locomotor activity. In the LD, vehicle-treated Foxp3-depleted male mice exhibited a significant increase in time spent in the dark compartment compared to vehicle-treated WT mice (Figure 1C; **p < 0.01). This phenotype was absent in vehicle-treated female Foxp3-depleted mice, which showed no divergence from vehicle-treated WT mice. BDPP treatment significantly reversed the anxiety-like phenotype in male mice, restoring values toward vehicle-treated WT levels (Figure 1C; ^###^p < 0.001), whereas no treatment effect was detected in females. In the OF, male mice showed no significant differences in locomotor activity across all four groups, indicating no detectable effect of genotype or treatment on locomotion. In contrast, both vehicle and BDPP-treated female Foxp3-depleted mice exhibited reduced locomotor activity compared to their corresponding WT groups, indicating an overall decrease in locomotion across Foxp3-depleted female groups (Figure 1D; **p < 0.01, ^§§^p < 0.01, ^§^p < 0.05). Collectively, these findings demonstrate that peripheral Tregs are necessary for the maintenance of affective and motivational homeostasis and identify BDPP as a potent modulator of this neurobehavioral axis.

### Peripheral Foxp3^+^ Tregs depletion induces inflammasome activation that is selectively attenuated by BDPP in males

**Figure 2.**
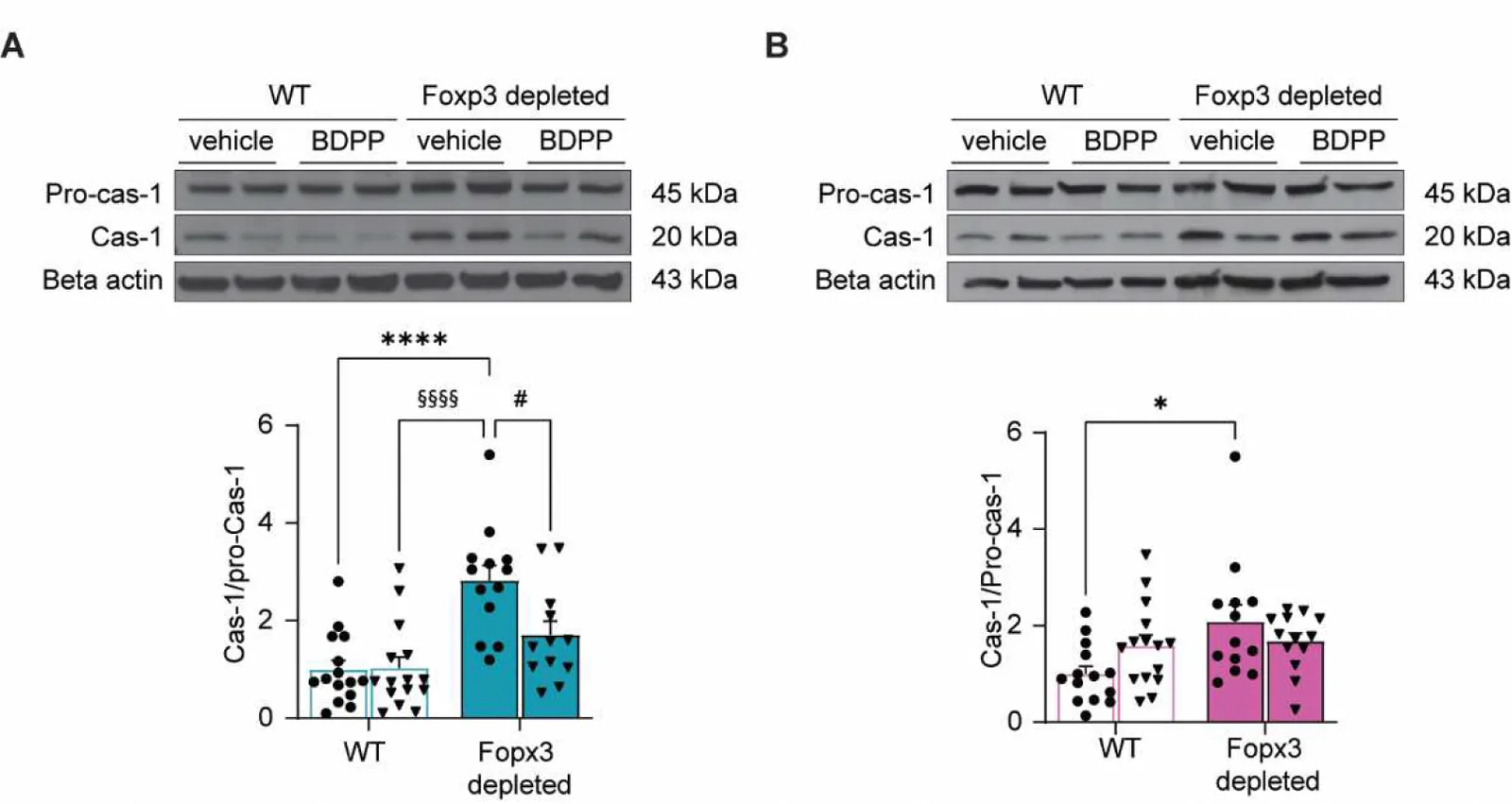
Hippocampal inflammasome activation following Foxp3^+^ Tregs depletion and its modulation by BDPP in male and female mice. (A) Representative western blots and quantitative analysis in male mice. Upper panel shows representative immunoblots of pro-cas-1 and cleaved cas-1, with β-actin as a loading control. Lower bar graphs show cleaved cas-1 levels and the cleaved cas-1/pro-cas-1 ratio as a measure of hippocampal inflammasome activation. (B) Parallel biochemical analyses in female mice. Upper panel shows representative western blots and lower bar graphs showing hippocampal inflammasome activation and the corresponding cleaved cas-1/pro-cas-1 ratio in the female cohort. WT groups are indicated by white bars with blue (males) or pink (females) borders, while Foxp3-depleted males and females are shown as solid blue and pink bars, respectively. Individual subjects are shown as circles (vehicle-treated) or inverted triangles (BDPP-treated). Data are presented as mean ± SEM. Statistical analyses were performed using two-way ANOVA followed by Tukey’s post hoc test (*p<0.05; ****p<0.0001; compared with age-matched vehicle-treated WT mice, ^§^p<0.05; ^§§§§^p<0.0001; compared with age-matched BDPP-treated WT mice, ^#^p<0.05; compared with age-matched vehicle-treated Foxp3-depleted mice, n = 12–15 mice for each group).

To assess whether the pro-inflammatory transcriptomic profile previously identified in male Foxp3-depleted mice translates into functional enzymatic activity (30), we quantified the cleavage of Caspase-1 (cas-1) in the hippocampus of both sexes. Consistent with our physiological observations (Supplementary Figure 1B), BDPP administration exhibited no discernible impact on cas-1 activation in WT mice, suggesting that BDPP does not interfere with baseline inflammasome signaling under homeostatic conditions (Figure 2A and 2B, Supplementary Figure 2). In male mice (Figure 2A), vehicle-treated Foxp3-depleted mice exhibited a significant increase in the cleaved cas-1 to pro-cas-1 ratio compared with vehicle-treated WT mice (****p<0.0001), indicating robust hippocampal inflammasome activation. Notably, under Foxp3-depleted conditions, BDPP treatment significantly attenuated this activation (^#^p<0.05), effectively suppressing the pro-inflammatory cascade in males. In female mice (Figure 2B), vehicle-treated Foxp3-depleted mice similarly displayed an elevated the cleaved cas-1 to pro-cas-1 ratio relative to vehicle-treated WT mice (*p<0.05), indicating that inflammasome activation also occurs in females. However, in contrast to the therapeutic efficacy observed in males, BDPP treatment failed to significantly attenuate cas-1 activation in Foxp3-depleted females. Taken together, these data demonstrate that peripheral Foxp3^+^ Tregs depletion induces hippocampal inflammasome activation in both sexes, whereas BDPP suppresses this pathological response exclusively in males, highlighting a sex-dependent therapeutic responsiveness to polyphenol intervention.

### Peripheral Foxp3^+^ Tregs depletion induces microglial hypertrophy that is ameliorated by BDPP in male

**Figure 3.**
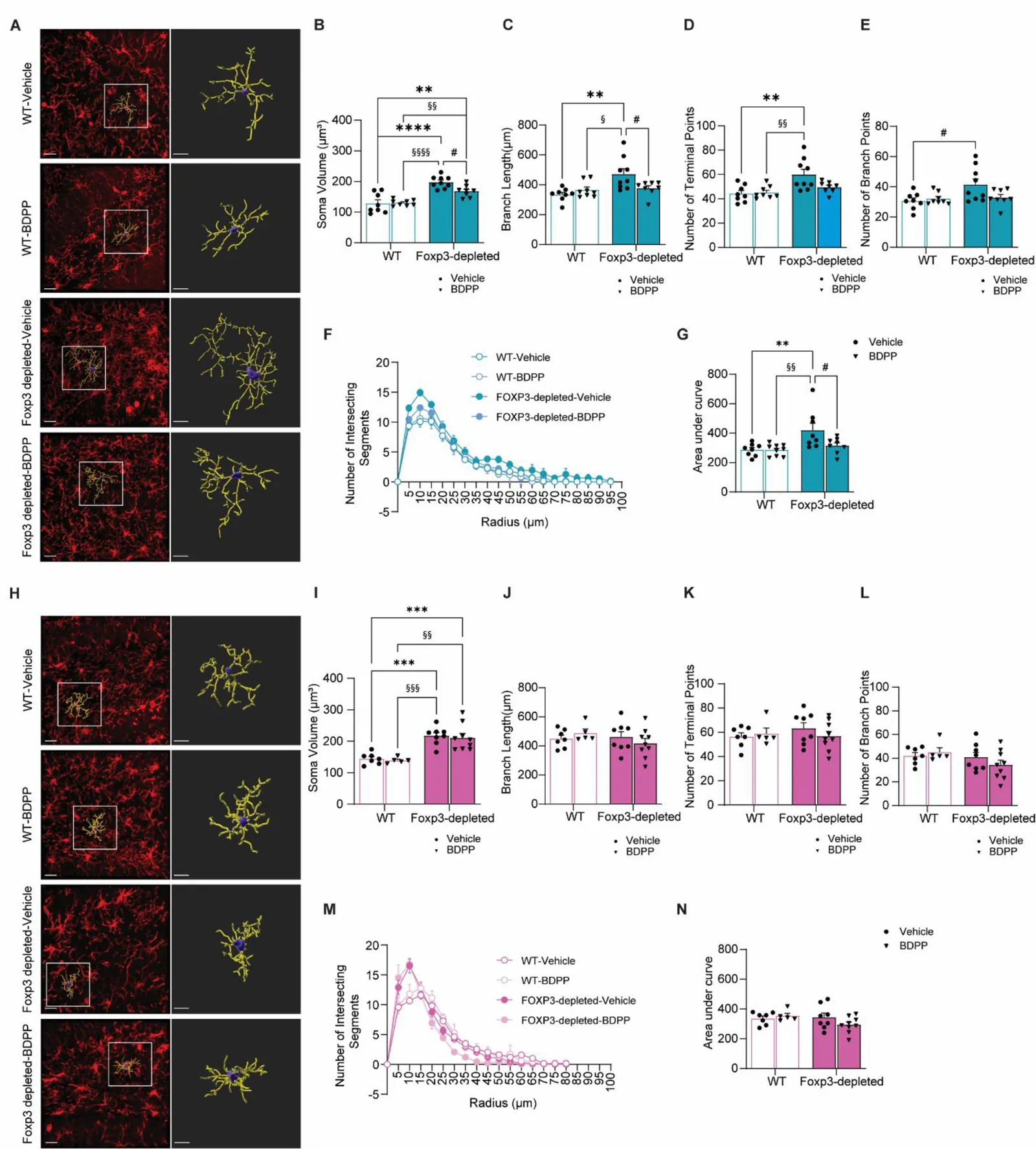
Sex-specific impact of BDPP on microglial morphological remodeling following Foxp3^+^ Tregs depletion. (A and H) Representative immunofluorescence images of IBA-1+ (red) microglia in the hippocampus of male (A) and female (H) mice. The area outlined with a white box in the left panels is magnified in the right panels. The right panels show representative three-dimensional (3D) reconstructions of microglial morphology. Scale bars: 20 um (left) and 10 um (right and middle). (B–G) Morphological quantification of hippocampal microglia in males. Bar graphs represent (B) soma volume, (C) total branch length, (D) number of terminal points, and (E) number of branch points. (F) Sholl analysis showing the number of intersections at increasing radii from the soma (5 um intervals). (G) Quantification of the Area Under the Curve for the Sholl analysis. (I–N) Morphological quantification of hippocampal microglia in females. Bar graphs represent (I) soma volume, (J) total branch length, (K) number of terminal points, and (L) number of branch points. (M) Sholl analysis showing the number of intersections at increasing radii from the soma (5 um intervals). (N) Quantification of the Area Under the Curve for the Sholl analysis. WT groups are indicated by white bars with blue (males) or pink (females) borders, while Foxp3-depleted males and females are shown as solid blue and pink bars, respectively. Individual subjects are represented as circles (vehicle-treated) or inverted triangles (BDPP-treated). Data are presented as mean ± SEM. Statistical analyses were performed using two-way ANOVA with repeated measures (*p<0.05; **p<0.01; ***p<0.001; compared with age-matched vehicle-treated WT mice, ^§^p<0.05; ^§§^p<0.01; ^§§§^p<0.001; ^§§§§^p<0.0001; compared with age-matched BDPP-treated WT mice, ^#^p<0.05; compared with age-matched vehicle-treated Foxp3-depleted mice, n = 3–5 mice for each group, with 4–15 microglia examined per section).

To investigate whether the robust hippocampal inflammasome activation correlates with structural changes in microglia, we assessed Sholl analysis and measured various morphological parameters. In male mice (Figure 3A–3G), vehicle-treated Foxp3-depleted mice displayed a distinct hypertrophic activation of microglia. This was characterized by a significant increase in soma volume (Figure 3B; ****p < 0.0001), along with an expansion in process architecture. Specifically, vehicle-treated Foxp3-depleted male mice exhibited significantly greater total branch length (Figure 3C; **p < 0.01), number of terminal points (Figure 3D; **p < 0.01), and branch points (Figure 3E; *p < 0.05) compared to vehicle treated WT-mice. Consistent with these architectural changes, Sholl analysis revealed a significant upward shift in the number of intersecting segments (Figure 3F and Table 1), resulting in corresponding increase in the area under the curve (AUC) (Figure 3G; **p < 0.01). Notably, BDPP administration in male selectively attenuated features of this microglial hypertrophy. While BDPP treatment significantly reduced soma volume (Figure 3B; ^§^p < 0.05) and restored the total branch length and Sholl AUC toward homeostatic levels (Figure 3C and 3G; ^§^p < 0.05), its effects on branching complexity were limited, as terminal points and branch points were not significantly altered (Figure 3D and 3E). Sholl analysis further indicated that BDPP primarily attenuated the increase in intersections within the intermediate radial range (5–10 μm), where the hypertrophic shift was most prominent (Figure 3F and Table 1).

In female mice (Figure 3H–3N), Foxp3 depletion produced a more restricted morphological response, characterized exclusively by a significant increase in soma volume(Figure 3I; ***p < 0.001), without detectable changes in total branch length, terminal points, or branch points (Figure 3J–3L). Consistently, Sholl analysis showed no significant differences across radial distances or AUC between groups (Figure 3M–3N and Table 1), indicating limited remodeling of microglial arborization in females following Foxp3 depletion. Furthermore, BDPP treatment did not significantly modify any morphological parameters in Foxp3-depleted females. Collectively, these data demonstrate that peripheral Treg depletion induces sex specific microglial phenotypes, and that BDPP selectively reverses microglia hypertrophy in males. These structural changes parallel the divergent patterns of hippocampal inflammasome activation and behavioral outcomes observed across sexes.

### Peripheral Foxp3^+^ Tregs depletion does not impair vascular integrity across sexes.

**Figure 4.**
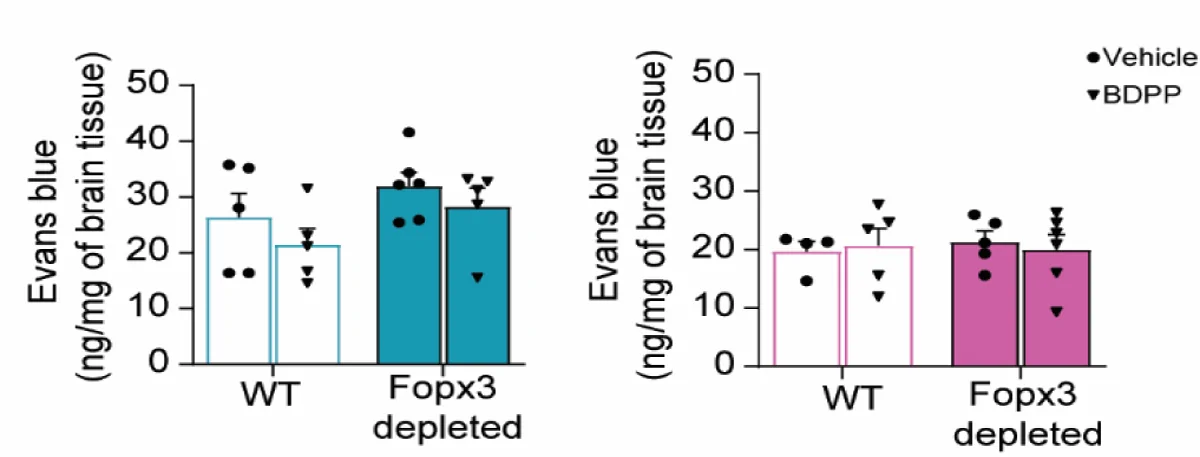
Maintenance of blood–brain barrier integrity following peripheral Foxp3^+^ Treg depletion and BDPP treatment. Bar graphs showing quantitative analysis of Evans Blue extravasation into the brain parenchyma, expressed as ng/mg tissue weight. WT groups are indicated by white bars with blue (males) or pink (females) borders, while Foxp3-depleted males and females are shown as solid blue and pink bars, respectively. Individual subjects are represented as circles (vehicle-treated) or inverted triangles (BDPP-treated). Data are presented as mean ± SEM. Statistical analyses were performed using two-way ANOVA followed by Tukey’s post hoc test (n = 4–6 mice for each group).

To examine whether the observed neuroinflammatory changes and BDPP-mediated effects are associated with alterations in BBB integrity, we assessed vascular permeability using the Evans Blue assay. In both male and female mice, peripheral Foxp3^+^ Treg depletion did not result in detectable Evans Blue extravasation into the brain parenchyma, with dye levels remaining comparable across all experimental groups (Figure 4). Furthermore, BDPP treatment had no significant effect on BBB permeability in either sex. Taken together, these data indicate that BBB integrity is preserved under all experimental conditions, suggesting that the observed sex-dependent effects are not attributable to differences in vascular permeability or drug access to the brain.

## Discussion

The present study identifies a peripheral immune–brain axis in which Foxp3^+^ Treg deficiency and BDPP jointly regulate hippocampal inflammasome activation, microglial remodeling, and affective behavioral outcomes. Importantly, these effects occurred in the absence of BBB disruption, indicating that peripheral immune imbalance and its pharmacological modulation influence central neuroimmune function through regulated signaling mechanisms rather than structural vascular compromise. This observation aligns with previous studies suggesting that peripheral immune signals can modulate brain function without overt BBB breakdown, potentially through neurovascular and immune interface mechanisms (37–39).

A central finding of this study is that BDPP selectively modulates distinct affective behaviors affected by Foxp3 depletion. Behavioral analyses revealed that Foxp3 depletion induces a coordinated neurobehavioral syndrome characterized by depressive-like behavior and impaired motivational drive, consistent with prior studies linking peripheral immune dysregulation to affective and motivational deficits (40, 41). Notably, although Foxp3 depletion induced these behavioral alterations in both sexes, the therapeutic effects of BDPP varied across behavioral domains and between males and females (Figure 1). BDPP more effectively improved affective and motivational deficits, whereas anxiety-related and locomotor phenotypes exhibited comparatively limited responsiveness. These findings suggest that BDPP does not uniformly normalize behavior function but rather selectively modulates neural systems.

The sex-dependent behavioral alterations observed across distinct behavioral domains likely reflect the interaction of multiple neurobiological mechanisms, including active coping regulation, neuroimmune signaling, and circuit-specific plasticity. First, sex hormones critically regulate synaptic plasticity and stress responsivity within hippocampal and prefrontal circuits, thereby shifting the functional set point of emotion-related neural networks (42–47). Second, microglia exhibit sex-specific developmental programming and transcriptional profiles that shape how peripheral immune signals are translated into central neuroimmune responses, influencing synaptic remodeling and inflammatory signaling (48–51). Third, affective and motivational behaviors arise from distributed and functionally dissociable neural circuits involving the hippocampus, prefrontal cortex, mesolimbic dopamine system, and amygdala, which differentially contribute to reward processing, cognitive control, and threat-related behavioral responses (52–55). Previous studies suggest that BDPP may differentially engage specific circuit nodes depending on sex-dependent baseline network states and plasticity capacity. In addition, accumulating evidence suggests that dietary polyphenols exert effects beyond classical anti-inflammatory actions, including modulation of synaptic plasticity and hippocampal function. Polyphenols have been shown to enhance long-term potentiation, dendritic spine density, and hippocampal-dependent cognitive processes, supporting a role in activity-dependent circuit remodeling (56–59). These findings support the interpretation that BDPP may influence behavior through modulation of neuroimmune–synaptic interactions rather than generalized immunosuppression. Furthermore, it is possible that the observed sex-dependent efficacy of BDPP stems from differential pharmacokinetic processing and bioavailability between sexes. Prior evidence indicates that the gastrointestinal metabolism of dietary polyphenols and the composition of the gut microbiota are highly sexually dimorphic (60, 61), which may lead to disparate systemic exposure to key brain-permeable metabolites, such as 3-hydroxyphenylpropionic acid and malvidin-glucoside (62). Consequently, variations in hepatic metabolic enzymes or microbial biotransformation could account for the attenuated therapeutic responsiveness observed in female mice (63). Collectively, these results suggest that BDPP acts as a modulator of sex-dependent neuroimmune interactions, rather than a generalized anti-inflammatory agent, with its behavioral effects reflecting differential engagement of neural systems underlying affective and motivational processes.

To define the upstream neuroimmune mechanisms linking peripheral Treg dysfunction to these cellular and behavioral alterations, we next examined hippocampal inflammasome signaling. In our previous study, transcriptomic profiling revealed significant enrichment of inflammasome-related pathways following Foxp3 depletion, indicating that peripheral immune perturbation induces selective activation of innate immune programs within the hippocampus (30). This finding is consistent with prior studies demonstrating that peripheral immune challenges can drive region-specific inflammatory transcriptional responses within limbic structures, including the hippocampus (64–66). In the present study, Foxp3-depleted mice exhibited increased cas-1 cleavage at the protein level in both males and females, indicating that inflammasome signaling is not limited to transcriptional priming but reflects a functional pathway of the activation (Figure 2). The inflammasome is a key molecular complex regulating IL-1β maturation and has been extensively implicated in the modulation of neuroinflammatory processes, synaptic plasticity, and neuronal excitability within the central nervous system (67). Microglia are considered the primary cellular mediators of this pathway, translating immune signals into circuit-level functional neuroimmune responses (9). Functionally, inflammasome activation and downstream IL-1β signaling have been linked to stress susceptibility, reward dysfunction, and depressive-like behavior, suggesting a mechanistic role in affective and motivational regulation (68). Together, these findings suggest that hippocampal inflammasome activation may contribute to affective and motivational abnormalities. Importantly, BDPP treatment significantly reduced cas-1 activation in males, whereas this effect was not observed in females, indicating a clear sex-dependent sensitivity of inflammasome regulation. This differential response suggests that biological sex is a critical determinant of how peripheral immune modulation translates into central inflammasome activity. Consistent with this interpretation, previous studies have shown that inflammasome signaling is subject to sex-dependent regulation, influenced by microglial developmental programming and sex hormone signaling pathways (69, 70). Collectively, these findings identify hippocampal inflammasome signaling as a key neuroimmune intermediate linking peripheral Foxp3^+^ Treg dysfunction to central immune activation, and demonstrate that BDPP modulates this pathway in a sex-dependent manner.

Consistent with the behavioral and inflammasome-related alterations observed following Foxp3 depletion, Sholl analysis revealed marked microglial remodeling within the hippocampus (Figure 3). While microglial soma size increased in both males and females, the spatial extent of microglial process arborization exhibited clear sex-dependent differences. In males, microglial processes extended to a greater radial distance, reaching approximately 95 μm, whereas in females process expansion was more limited, terminating at approximately 80 μm. This indicates that Foxp3 depletion differentially alters the effective microglial surveillance territory across biological sex. Such differences in radial extent suggest that male microglia survey a broader hippocampal microenvironment, potentially influencing local cellular interactions across a broader spatial domain. In contrast, the more restricted spatial reach observed in females may reflect a constrained surveillance field under conditions of peripheral immune dysregulation. Given that microglial process dynamics are tightly coupled to synaptic monitoring and circuit refinement (71), these structural differences may contribute to sex-dependent differences in hippocampal neuroimmune states. These structural alterations are consistent with the sex-dependent behavioral phenotypes observed in the present study. Male Foxp3-depleted mice exhibited both anxiety and depressive-like behavioral deficits, whereas females primarily displayed depressive-like behaviors without prominent anxiety-related phenotypes. Given that microglial process dynamics are closely linked to synaptic surveillance and neuronal circuit modulation (9, 71), the enhanced arborization observed in males may reflect altered microglial interactions within hippocampal cellular networks under conditions of chronic immune activation. In contrast, the more limited microglial remodeling observed in females may indicate a distinct structural response to immune dysregulation that is less strongly associated with anxiety-related behavioral phenotypes. Importantly, these microglial changes occurred in parallel with increased cas-1 cleavage, supporting a close association between inflammasome activation and microglial structural remodeling. Previous studies have demonstrated that inflammasome-associated signaling pathways can directly regulate microglial morphology, process motility, and inflammatory responsiveness (72). In addition, chronic neuroimmune activation has been shown to alter microglia–synapse interactions and disrupt neuronal circuit stability, particularly within stress-sensitive limbic regions (73). Importantly, BDPP treatment selectively normalized the hyper-ramified morphology observed in males, reducing both soma enlargement and excessive branching complexity toward control levels. This structural normalization closely paralleled the suppression of cas-1 activation and the improvement of affective and motivational behaviors, suggesting parallel changes in inflammasome signaling, microglial state, and behavioral outcomes. Collectively, these findings support a model in which peripheral Foxp3 depletion promotes sex-dependent neuroimmune remodeling within the hippocampus, linking inflammasome activation and altered microglial structure to behavioral dysfunction.

Importantly, vascular integrity, as assessed by the absence of Evans Blue extravasation, was preserved across all experimental groups (Figure 4). This indicates that neither Foxp3 depletion nor BDPP treatment significantly affects blood–brain barrier permeability under the present experimental conditions. Accordingly, the observed behavioral and neuroimmune changes are unlikely to result from nonspecific peripheral immune cell infiltration or differences in central exposure. These findings support a model in which peripheral immune dysregulation influences central nervous system function through signaling mechanisms that operate across an intact blood–brain barrier. In this context, the neurovascular unit and the meningeal lymphatic–cerebrospinal fluid system represent plausible routes for immune–brain communication. These interfaces have been shown to mediate the exchange of immune and metabolic signals between the periphery and the brain without disruption of barrier integrity (74, 75). Consequently, the therapeutic actions of BDPP are likely mediated through the modulation of these endogenous neuroimmune interfaces, rather than the restoration of a disrupted barrier or the restriction of peripheral infiltration. These results are consistent with the notion that BDPP exerts its behavioral and neuroinflammatory effects within a structurally intact neurovascular framework.

Cumulative pharmacokinetic evidence indicates that bioactive polyphenol metabolites derived from BDPP possess a distinct bioavailability profile that enables them to cross the BBB and accumulate within the central nervous system, even under physiological conditions where the neurovascular unit remains intact (22, 58). Consequently, the capacity of BDPP to modulate microglial morphology and suppress cas-1 activation observed in the present study is firmly supported by this established pharmacokinetic framework. This CNS permeability strongly suggests that BDPP may directly regulate neuroimmune responses within the hippocampal microenvironment, potentially targeting the sex-dependent divergence in inflammatory signaling cascades induced by Foxp3^+^ Treg deficiency. Previous studies have demonstrated that male microglia exhibit a higher baseline sensitivity toward NF-κB-associated inflammatory priming than their female counterparts (48, 76), providing a robust molecular rationale for the male-predominant therapeutic responses delineated herein. While these findings point to the involvement of BDPP in modulating upstream inflammatory priming signals, the current data do not warrant a definitive conclusion as to whether its molecular targets are differentially regulated by sex in a direct manner. Nonetheless, the present study clearly demonstrates in vivo that BDPP distinctively attenuates peripheral immune disruption-driven alterations in hippocampal neuroimmunity and microglial architecture in a sex-specific manner. Thus, these findings suggest that BDPP does not act as a nonspecific systemic immunosuppressant, but rather functions as a multi-pathway immunomodulator capable of modulating the peripheral–central neuroimmune axis. These data underscore the importance of developing precision neuroimmunotherapy strategies with potential clinical relevance that account for sex-dependent differences in therapeutic efficacy.

Several limitations of the present study should be acknowledged. First, although our findings demonstrate a robust association between peripheral Tregs dysfunction and hippocampal inflammasome activation, the upstream mechanisms linking peripheral immune perturbation to central immune responses remain unresolved. Multiple non-mutually exclusive pathways may contribute to this communication, including cytokine-mediated signaling, circulating immune-derived factors, metabolic changes, and neurovascular or meningeal interfaces. Further studies will be required to delineate the relative contribution of these pathways. In particular, given the absence of overt BBB disruption in both sexes, future investigations should clarify whether peripheral immune signals access the brain through regulated transport mechanisms or endothelial-mediated signaling pathways, rather than passive paracellular diffusion. Second, although BDPP consistently attenuated both behavioral and molecular phenotypes, the present data do not allow definitive conclusions regarding its primary site or mode of action. BDPP may act through the modulation of peripheral immune function, indirect effects on central immune signaling, or integrated actions across multiple biological compartments. Accordingly, BDPP is best interpreted as a modulator of the broader immune–brain axis rather than a pathway- or cell-type–specific intervention. Third, although the hippocampus was selected as the primary region of interest due to its established role in affective regulation and immune integration, the involvement of additional brain regions cannot be excluded. Given the systemic nature of Foxp3-associated immune alterations, coordinated adaptations across corticolimbic and midbrain circuits are likely to contribute to the observed behavioral phenotypes. Future studies employing spatial transcriptomics and circuit-level analyses will be necessary to define the full anatomical organization of this immune–brain network.

In conclusion, Foxp3^+^ Treg deficiency induced hippocampal inflammasome activation, microglial morphological remodeling, and sex-dependent behavioral alterations. BDPP partially reversed these molecular and behavioral changes, with stronger effects in males. These findings identify BDPP as a modulator of neuroimmune and behavioral outcomes associated with peripheral immune dysregulation, with effects that are modulated in a sex-dependent manner.

## Supplementary Material

Supplement 1

## Data Availability

The datasets generated during and/or analyzed during the current study are available from the corresponding author on reasonable request.
