# Supplementary material for: Bioactive dietary polyphenols modulate hippocampal inflammasome activation and sex-specific affective behavioral deficits following peripheral Foxp3+ regulatory T cell depletion
Source: Res Sq. 2026 Aug 24:rs.3.rs-10425197. Preprint. [Version 1] doi: 10.21203/rs.3.rs-10425197/v1 (PMC13532728; doi:10.21203/rs.3.rs-10425197/v1)
Supplement: Supplementary file 1 [file NIHPPRS10425197v1-supplement-1.pdf]

Supplementary Figure 1

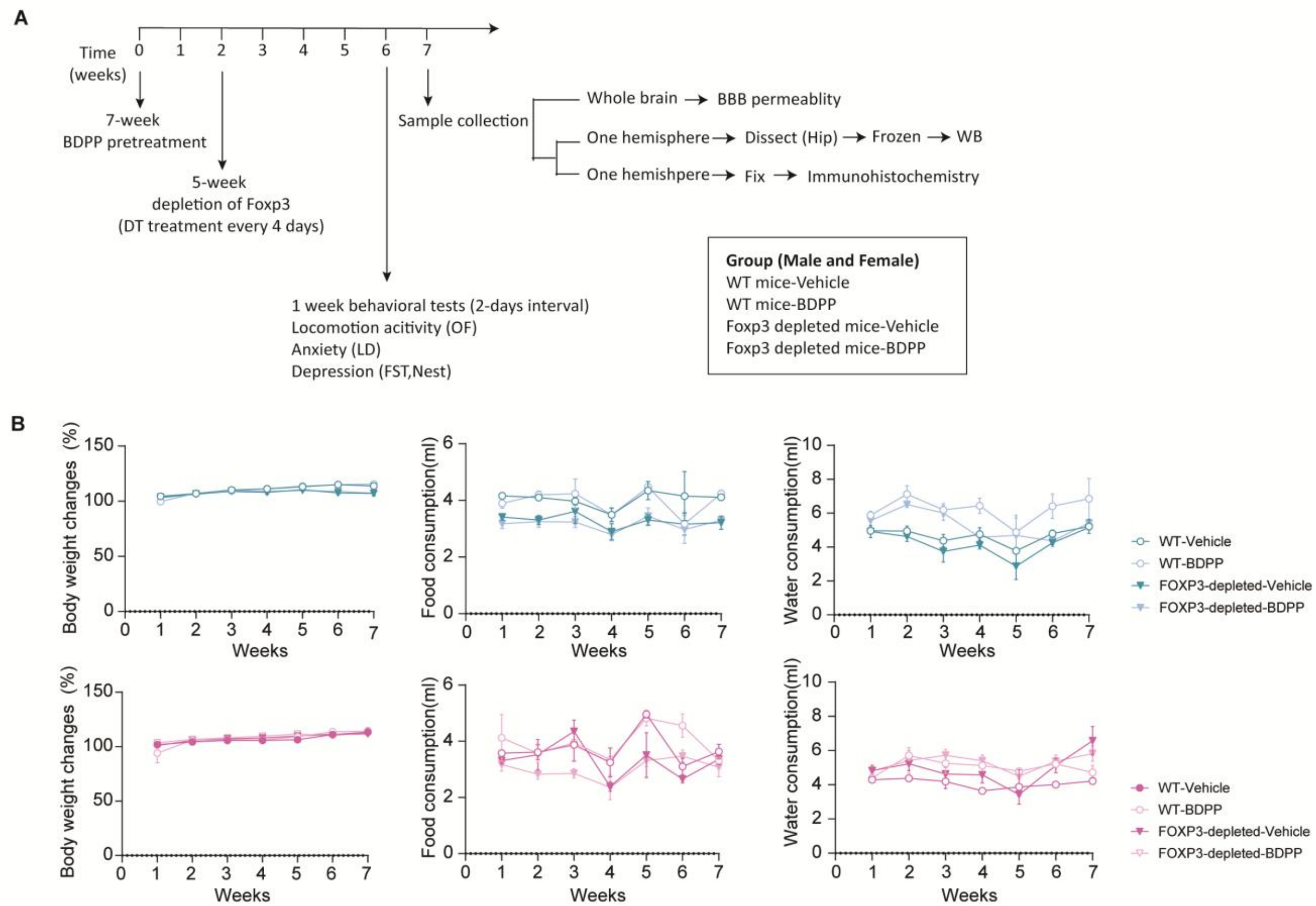

Supplementary Figure 1. Experimental design and longitudinal physiological monitoring.

(A) Schematic overview of the experimental timeline. Mice received a 7-week pretreatment with BDPP administration beginning at Week 0. At Week 2, depletion of Foxp3^{+} Treg was initiated by DT administration every 4 days and maintained for 5 weeks. Behavioral assessments were conducted during Week 6 and included the OF, LD, FST, and nest-building test. At Week 7, tissue collection was performed for analyses of BBB permeability, hippocampal protein expression by western blot (WB), and immunohistochemistry (IHC). (B) Longitudinal assessment of basal physiological parameters in male and female mice. Body weight, food intake, and water consumption were measured weekly throughout the study to evaluate potential systemic effects of BDPP treatment and Foxp3 depletion. Experimental groups are indicated in the graph. Data are presented as mean ± SEM. Male and female cohorts are represented in blue and pink, respectively. Symbols denote experimental groups as indicated in the graph.

Supplementary Figure 2

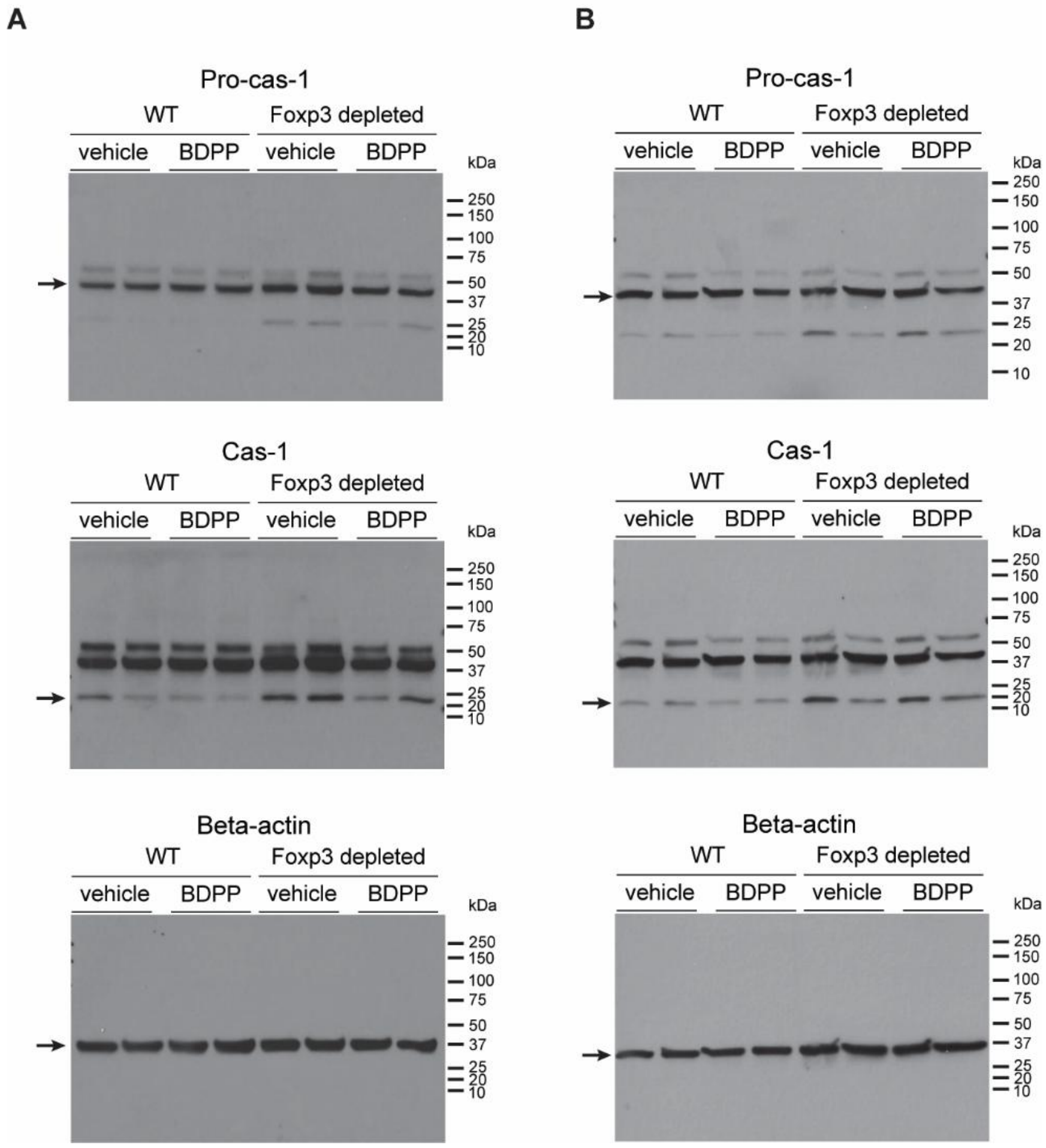

Supplementary Figure 2. Full-length uncropped western blot images.

(A) Original full-length blots for male mice. (B) Original full-length blots for female mice. In each panel, top cropped images correspond to pro-caspase-1 (Pro-cas-1), middle images represent cleaved caspase-1 (Cas-1), and bottom images indicate beta-actin, which served as a loading control. Arrows indicate the cropped areas displayed in the main figures.

Male	1	2	3	4	5	6	7	8	9	10	11	12	13	14	15	16	17	18	19	20
WT-Vehicle vs WT-BDPP	na	ns	ns	ns	ns	ns	ns	ns	ns	ns	ns	ns	ns	ns	na	ns	ns	ns	ns	ns
WT-Vehicle vs Foxp3-depleted-Vehicle	na	*	***	*	ns	ns	ns	ns	ns	*	ns	ns	ns	ns	ns	ns	na	na	na	na
WT-Vehicle vs Foxp3-depleted-BDPP	na	ns	*	ns	ns	ns	ns	ns	ns	ns	ns	ns	ns	ns	na	ns	ns	ns	ns	ns
WT-BDPP vs Foxp3-depleted-Vehicle	na	**	***	*	ns	ns	ns	ns	ns	*	ns	ns	ns	ns	ns	ns	na	na	na	na
WT-BDPP vs Foxp3-depleted-BDPP	na	ns	*	ns	ns	ns	ns	ns	ns	ns	ns	ns	ns	ns	na	ns	ns	ns	ns	ns
Foxp3-depleted-Vehicle vs Foxp3-depleted-BDPP	na	ns	*	ns	ns	ns	ns	ns	ns	ns	ns	ns	ns	ns	ns	na	na	na	na	na

Female	1	2	3	4	5	6	7	8	9	10	11	12	13	14	15	16	17
WT-Vehicle vs WT-BDPP	na	ns	ns	ns	ns	ns	ns	ns	ns	ns	ns	ns	ns	ns	ns	ns	ns
WT-Vehicle vs Foxp3-depleted-Vehicle	na	ns	**	ns	ns	ns	ns	ns	ns	ns	ns	ns	ns	ns	ns	ns	ns
WT-Vehicle vs Foxp3-depleted-BDPP	na	ns	***	ns	ns	**	**	**	***	**	ns	*	*	ns	ns	ns	ns
WT-BDPP vs Foxp3-depleted-Vehicle	na	ns	*	ns	ns	*	ns	ns	ns	ns	ns	ns	ns	ns	na	ns	ns
WT-BDPP vs Foxp3-depleted-BDPP	na	ns	**	ns	*	***	*	*	**	*	ns	ns	ns	ns	na	ns	ns
Foxp3-depleted-Vehicle vs Foxp3-depleted-BDPP	na	ns	ns	ns	ns	ns	**	*	ns	ns	ns	ns	ns	ns	na	na	na

Table 1. Statistical analysis of microglial Sholl intersections in male and female mice.

Detailed p-values across all radii. Statistical significance was determined by two-way ANOVA with repeated measures (RM) followed by Tukey's post hoc test for multiple comparisons. The numbers in the first row represent the distance from the soma in 5 um increments, where '1' corresponds to 0 um and '20' corresponds to 95 um. Significant differences between groups are indicated as *p < 0.05, **p < 0.01, ***p < 0.001, and ****p < 0.0001, while ns denotes not significant and na indicates not applicable due to radii with zero or insufficient intersections for analysis.

Supplementary Files

This is a list of supplementary files associated with this preprint. Click to download.

- Table1.xlsx
- SupplementaryInfoFile.pdf
